# Sensor-Controlled Digital Game for Heart Failure Self-management: Protocol for a Randomized Controlled Trial

**DOI:** 10.2196/45801

**Published:** 2023-05-10

**Authors:** Kavita Radhakrishnan, Christine Julien, Matthew O'Hair, Rachel Tunis, Grace Lee, Angelica Rangel, James Custer, Tom Baranowski, Paul J Rathouz, Miyong T Kim

**Affiliations:** 1 School of Nursing The University of Texas at Austin Austin, TX United States; 2 Department of Electrical and Computer Engineering University of Texas at Austin Austin, TX United States; 3 Good Life Games, LLC Austin, TX United States; 4 School of Information University of Texas at Austin Austin, TX United States; 5 Department of Population Health Dell Medical School The University of Texas at Austin Austin, TX United States; 6 Children's Nutrition Research Center Department of Pediatrics Baylor College of Medicine Houston, TX United States

**Keywords:** heart failure, digital game, self-management, mobile phone, older adults

## Abstract

**Background:**

Heart failure (HF) is the leading cause of hospitalization among older adults in the United States. There are substantial racial and geographic disparities in HF outcomes, with patients living in southern US states having a mortality rate 69% higher than the national average. Self-management behaviors, particularly daily weight monitoring and physical activity, are extremely important in improving HF outcomes; however, patients typically have particularly low adherence to these behaviors. With the rise of digital technologies to improve health outcomes and motivate health behaviors, sensor-controlled digital games (SCDGs) have become a promising approach. SCDGs, which leverage sensor-connected technologies, offer the benefits of being portable and scalable and allowing for continuous observation and motivation of health behaviors in their real-world contexts. They are also becoming increasingly popular among older adults and offer an immersive and accessible way to measure self-management behaviors and improve adherence. No SCDGs have been designed for older adults or evaluated to test their outcomes.

**Objective:**

This randomized clinical trial aims to assess the efficacy of a SCDG in integrating the behavioral data of participants with HF from weight scale and activity tracker sensors to activate game progress, rewards, and feedback and, ultimately, to improve adherence to important self-management behaviors.

**Methods:**

A total of 200 participants with HF, aged ≥45 years, will be recruited and randomized into 2 groups: the SCDG playing group (intervention group) and sensor-only group (control group). Both groups will receive a weight scale, physical activity tracker, and accompanying app, whereas only the intervention group will play the SCDG. This design, thereby, assesses the contributions of the game. All participants will complete a baseline survey as well as posttests at 6 and 12 weeks to assess the immediate effect of the intervention. They will also complete a third posttest at 24 weeks to assess the maintenance of behavioral changes. Efficacy and benefits will be assessed by measuring improvements in HF-related proximal outcomes (self-management behaviors of daily weight monitoring and physical activity) and distal outcomes (HF hospitalization, quality of life, and functional status) between baseline and weeks 6, 12, and 24. The primary outcome measured will be days with weight monitoring, for which this design provides at least 80% power to detect differences between the 2 groups.

**Results:**

Recruitment began in the fall of 2022, and the first patient was enrolled in the study on November 7, 2022. Recruitment of the last participant is expected in quarter 1 of 2025. Publication of complete results and data from this study is expected in 2026.

**Conclusions:**

This project will generate insight and guidance for scalable and easy-to-use digital gaming solutions to motivate persistent adherence to HF self-management behaviors and improve health outcomes among individuals with HF.

**Trial Registration:**

ClinicalTrials.gov NCT05056129; https://clinicaltrials.gov/ct2/show/NCT05056129

**International Registered Report Identifier (IRRID):**

DERR1-10.2196/45801

## Introduction

### Background

More than 6 million adults in the United States live with heart failure (HF), a condition that is the leading cause of hospitalization among older adults in the United States [1]. Despite substantial advances in treatment and management, the current estimated annual cost is US $32 billion, which is projected to rise to US $69 billion by 2030 [2]. There are major regional disparities among individuals diagnosed with HF in the southern United States, with a mortality rate 69% higher than the national average [3,4].

Self-management behaviors are important for patients with HF to help reduce serious adverse sequelae and outcomes [5]. For example, weight gain is an initial warning sign of volume overload in patients with HF; by monitoring their weight daily, patients may receive prompt treatment for their weight gain, which can help avoid clinically significant exacerbations (odds ratio 0.42, 95% CI 0.23-0.76) [6,7]. Similarly, physical activity is linked to clinically significant improvements, such as improved myocardial function (*P*=.02) and functional capacity (*P*<.001) [8] and reduced depressive symptoms [9]. By self-managing these 2 critical elements, patients with HF experienced improved quality of life (QoL) and reduced health care use and cost in high-quality research studies [10,11]. Conversely, poor HF self-management may delay recognition of severe HF symptoms and result in more frequent hospitalizations, impaired functional status, and poorer QoL [12].

Despite its importance, persistent adherence to critical self-management behaviors continues to remain a challenge for patients with HF [13,14]. Patients have poorer adherence to weight monitoring and physical activity than to other self-management behaviors [15]. Thus, there is a need for novel approaches to support and motivate patients with HF to self-manage these critical behaviors. Interventions that are remote, resource conservative, and can be used in home may help address the widening geographical [3,4] and racial [16] health disparities in HF outcomes. Wearable technologies, mobile health apps, and sensor devices offer substantial promise in that they are portable, scalable, and allow for continuous observation and motivation of health behaviors in real-world contexts. Despite these benefits, the adoption of these technologies is low; abandonment is common [17], and longitudinal studies, especially in the context of chronic health conditions, are rare. Although the potential of wearables and digital sensor technologies for HF to improve care and outcomes has been acknowledged [18] and proven [19,20], the studies exploring their use in practice have largely been limited in size and scale [21].

### Objectives

One promising approach to increasing adherence to self-management behaviors in patients with HF is to leverage the use of digital technologies such as sensor-controlled digital games (SCDGs). SCDGs synchronize data on behaviors from sensors, such as wearable devices, with a mobile gaming app, allowing game progress, rewards, incentives, and personalized feedback (eg, reduce fluid intake or contact a physician) based on real-time contextual information (eg, weight gain or concerning symptoms) [22]. To promote change and its maintenance, SCDGs can also incorporate immersive stories [23], active learning [24], and social connections [25]. SCDGs are not only affordable, portable, and scalable, but they are also enjoyable and easy to use. Furthermore, prior research has indicated that they can also contextualize health behaviors [26], motivate behavioral changes, and improve health outcomes [27]. Thus, SCDGs offer an immersive and accessible way to objectively measure self-management behaviors and improve adherence [28]. Our previous research [29] conducting an SCDG intervention with 38 adults (aged ≥55 years) with HF from Texas and Oklahoma demonstrated improvements in weight monitoring and physical activity, and the intervention showed a high rate of acceptability (92%) from participants.

In this clinical trial, we have updated the SCDG intervention and will evaluate its efficacy for improving HF outcomes by integrating behavioral data of participants with HF from weight scale and activity tracker sensors to activate game progress, rewards, and feedback.

## Methods

### Study Design

This study is an efficacy clinical trial, using a prospective, 2-group randomized (1:1) controlled design in which an SCDG playing group with sensors and tracking apps (intervention group [IG]) will be compared with a sensor-only group (control group [CG]) that receives only the app that tracks weight monitoring and physical activity. In a sample of 200 individuals with HF, who are aged ≥45 years (n=100 per arm), benefits will be assessed by measuring improvements in HF-related proximal outcomes (self-management behaviors of daily weight monitoring and daily physical activity) and distal outcomes (HF hospitalization, QoL, and functional status) between baseline and weeks 6, 12, and 24. We will also apply mixed methods to assess IG and CG participants’ perceptions of intervention and behavior adherence.

### Study Setting

We will enroll 200 patients in this study, 100 each in the IG and CG. To recruit patients, we are contracting with Trialfacts [30], a web-based recruiting company. Participants will be recruited from 7 southern states of the United States to address prominent geographic health disparities in HF [3,4]. These states are Alabama, Arkansas, Georgia, Louisiana, Mississippi, Oklahoma, and Texas, where people experience higher HF rates than the rest of the country.

### Eligibility Criteria

Participants must be aged ≥45 years (lifetime risk for HF is high for the age group of 45-95 years) [1]; have been hospitalized for HF in the last 12 months (to reduce the variance in recovery from a recent HF hospitalization); and be classified as class I, II, or III per the New York Heart Association’s HF classes [31] (thus ensuring recruitment of those who would benefit the most from self-management interventions). They must also be fluent in English, able to pass a mini–cognitive screening assessment [32], and able to walk independently without using a walker or other assistance. Exclusion criteria include (1) having severe visual or tactile impairments (eg, legal blindness or severe arthritis), which might make using smartphones and sensor devices difficult; (2) having undergone a heart transplantation or implantation of a durable mechanical circulatory support device in the past; (3) having a history of renal failure (which adversely affects HF prognosis); or (4) being diagnosed with an end-stage or terminal illness (eg, cancer). The study is registered at ClinicalTrials.gov (NCT05056129).

### Ethics Approval and Informed Consent

The protocol was approved by the Institutional Review Board of the University of Texas, Austin (STUDY00001438), on February 18, 2022. Informed consent will be obtained from all eligible participants through a phone call before baseline surveys are sent and data collection begins. After a member of the research team reads the consent form and solicits questions, participants will receive the consent form via email and be requested to provide their digitally signed consent form through the REDCap (Research Electronic Data Capture; Vanderbilt University) web-based survey database. Participants who prefer to sign a physical copy will be mailed a consent form. This approach will allow participants sufficient time to follow-up with questions and discuss the study with their family or friends before officially signing the consent form. Our team will make it clear to participants that they may decline to answer specific questions or terminate their involvement in the study at any time.

### Enrollment and Randomization Procedures

Participants will be assigned to either the IG or CG through computer-automated randomization. These assignments will be stratified based on sex (male, female, or other) and age (45-64 years or ≥64 years) to ensure balance in the study design. The allocation sequence for permuted block randomization with random block sizes of 4 and 6 was created by a statistical analyst who is not connected to the participant enrollment process. The allocation sequence will be uploaded to REDCap, which will automatically assign the participants to groups when they are enrolled in the study. The research team will be blinded to assignment group or maintained in a way that ensures the separation of intervention implementation from evaluation, thus reducing any potential bias (eg, ascertainment bias) in the collection and analysis of outcome data. For example, data collectors will not be a part of the intervention delivery team. Similarly, participants will be blinded to their group assignment, as both groups use digital tools. All participants will be provided with the same informed consent form that includes general terms such as *digital health tools* to enable blinding of participants to their group assignment.

### Intervention Description

**Figure 1:**
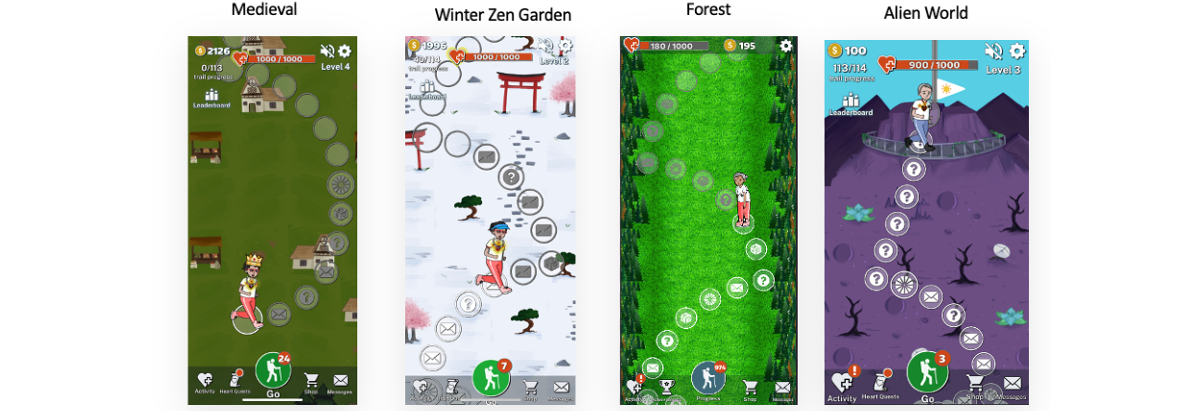
Screenshots from the different interfaces that make up the different levels within the sensor-controlled digital game.

The SCDG, which will be used by the IG in this study, is a smartphone app called Heart Health Mountain, which is available on both iOS and Android platforms. The design of the game was guided by behavioral change mechanisms using the concepts of motivation, ability, and trigger from the Fogg behavioral model and self-determination theoretical frameworks, as described in detail in a previous study [33]. This app has already been developed by a multidisciplinary team and tested through initial usability and feasibility studies and is described in detail elsewhere [28,29]. Several screenshots from the SCDG are pictured in Figure 1.

The SCDG fosters relatedness by involving a narrative, the goal of which is to help the game avatar avoid rehospitalization using game points, earned via the participants’ demonstration of competence or ability in real-time behaviors, in game tasks that help maintain the avatar’s optimal HF health status. The participants’ real-time behaviors of weight monitoring and physical activity will be tracked by off-the-shelf sensors and an app (Withings Pulse HR tracker, Body scale, and Health Mate app; Withings app). The data from the Withings sensors will then be routed to our SCDG app.

The digital game paired with sensors will enable objective tracking of real-time behaviors such as physical activity and weight monitoring and provide personalized, contextually relevant feedback (eg, reduce fluid intake or call a physician for weight gain) as triggers to motivate engagement in and generate habit formation of HF-related self-management behaviors. The game is adaptive to real-time behaviors; for example, participants’ step goals automatically adapt each week to encourage a tolerable physical activity level based on a rank-ordered algorithm [34].

### Control Description

The CG will use the same Withings devices over the same period but will not have access to the SCDG. Real-time behaviors of weight monitoring and physical activity will be tracked by the Withings Pulse heart rate tracker, Body scale, and Health Mate app. However, the data from the Withings sensors will not be routed to the SCDG. Instead, the CG will receive the same educational content that is presented in the SCDG in a paper format by mail. This study design feature will allow us to isolate the SCDG effect from the other features of behavioral motivation.

### Remote Study Procedures

All research activities will be conducted at participants’ homes. Participants will be directed to engage in research activities through remote means (email, phone call, or video calls). Participants will be asked to provide a convenient time for a research team member to contact them.

Participants in both the IG and CG will receive their Withings devices by mail, along with information on how to install the HealthMate app and connect the devices. The IG will also receive instructions on how to install the SCDG app (Table 1).

**Table 1:** Components of interventions received by the intervention group (IG) and control group (CG) participants.

Devices and apps	IG	CG
Withings weight scale and activity tracker	✓	✓
Withings HealthMate app for sensor data tracking	✓	✓
SCDG^a^ app with progress of game, messages, and incentives tailored to participant’s real-time HF^b^ self-management behaviors	✓	
Standard evidence-based HF educational content [35,36] embedded in SCDG	✓	
Standard evidence-based HF educational content [35,36] provided in paper format		✓

^a^SCDG: sensor-controlled digital game.

^b^HF: heart failure.

Participants in both groups will receive remote training from a member of the study team on how to install and use the devices. This remote training (ie, over Zoom [Zoom Video Communications] or FaceTime [Apple iOS]) was also successfully provided in our feasibility study [29]. After 3 days, both the groups will receive a follow-up email from the interventionist to summarize participants’ study tasks and provide contact information in case of troubleshooting needed while using the devices and apps.

Both groups will use the devices for 24 weeks. A dashboard was developed to track the time stamps of behavior data that were last synced by the participants to identify participants without any behavior data for >3 days. On a different page, the dashboard also shows the time stamps of the last time each participant in the IG opened the SCDG app. An unblinded team member aware of troubleshooting procedures and following a protocol to communicate similarly with participants in both groups will reach out to participants in both groups who have not had any behavior data from their devices synced in 3 days. This member would follow-up with participants in both groups daily for 3 business days. In case of no response, they will reach out to the alternative contact person listed by the participant.

### Randomized Controlled Trial Data Collection

All the survey data will be collected using the REDCap web-based survey system. After completing the baseline survey, all participants will complete the first and second posttests at 6 and 12 weeks for assessment of the *immediate effect of the intervention* and will complete the *third* posttest at 24 weeks after the first week for assessment of the *maintenance of behavioral changes*. Survey links will be sent via text messages to participants at 4 time points through automated events programmed in the REDCap survey system. In addition, daily sensor data will be obtained from the Withings server, and the game playing data will be obtained from a backend server hosted on Amazon Web Services. The steps, weight, and game state data will also be stored in a MongoDB database. All variables will be measured at all follow-ups using the instruments described in detail in Table 2.

**Table 2:** Data collected and operationally defined, with time points.

Variables		Instrument or measure	Time point (weeks)
**Baseline characteristics**			
	Sociodemographic	Sociodemographic and Upstream Risk Questionnaire [37,38]	Baseline
	Clinical	Hospital discharge summary (medications, comorbidities, and ejection fraction)	Baseline
	Weight monitoring frequency in the past week	1-item question using Likert scale	Baseline
	Depressive symptoms	Patient Health Questionnaire-2 [39,40]^a,b^	Baseline and 6, 12, and 24
	Ability		
	HF^c^ self-management knowledge	30-item Atlanta Heart Failure Knowledge Test (Cronbach α=.84) [41]^d^	Baseline and 6, 12, and 24
	HF self-efficacy	6-item Section C Self-Care of HF Index (Cronbach α=.88) [42]^d^	Baseline and 6, 12, and 24
**Trigger**			
	Engagement with SCDG^e^ and features	Backend game state logs of IG^f^ participants; Intrinsic Motivation Inventory [43] and Qualitative Interviews	6, 12, and 24; 12
**Motivation or behavior outcomes**			
	Number of days of weight monitoring (primary)^g^	HealthMate app logs [44] in IG and CG^h^	6, 12, and 24
	Daily physical activity steps	HealthMate app logs [44] in IG and CG	6, 12, and 24
	Daily physical activity intensity (light, moderate, or intense)	HealthMate app logs [44] in IG and CG	6, 12, and 24
	HF self-management behaviors	9-item European Heart Failure Self-care Behavior Scale (Cronbach α=.80) [45]^b,d^	Baseline, 6, 12, and 24
**Patient outcomes**			
	Functional status	23-item Kansas City Cardiomyopathy Questionnaire [46]^b,d^; functional status (Cronbach α=.93) [46]; QoL^i^ (Cronbach α=.78) [46]	Baseline and 6, 12, and 24
	QoL	Participant self-report	Baseline and 6, 12, and 24
	HF hospitalization	Participant self-report and confirmed through hospitalization discharge summary and health care provider	Baseline and 6, 12, and 24

^a^A total of 90% sensitivity in patients with HF and reliable prognostic prediction of mortality and rehospitalization.

^b^Established validity using correlation with other instruments or clinical ratings in known groups.

^c^HF: heart failure.

^d^Established reliability using interitem correlations and test-retest. Instruments take 30-55 minutes to complete when administered together.

^e^SCDG: sensor-controlled digital game.

^f^IG: intervention group.

^g^Outcome measure.

^h^CG: control group.

^i^QoL: quality of life.

The incentive of a gift card worth US $10 will be provided for the completion of surveys at each data collection time point (baseline and 6, 12, and 24 weeks) through Tango, a digital gift card disbursement system [47], as a reward for their effort. All participants will retain the scale and tracker till the end of the study.

**Figure 2:**
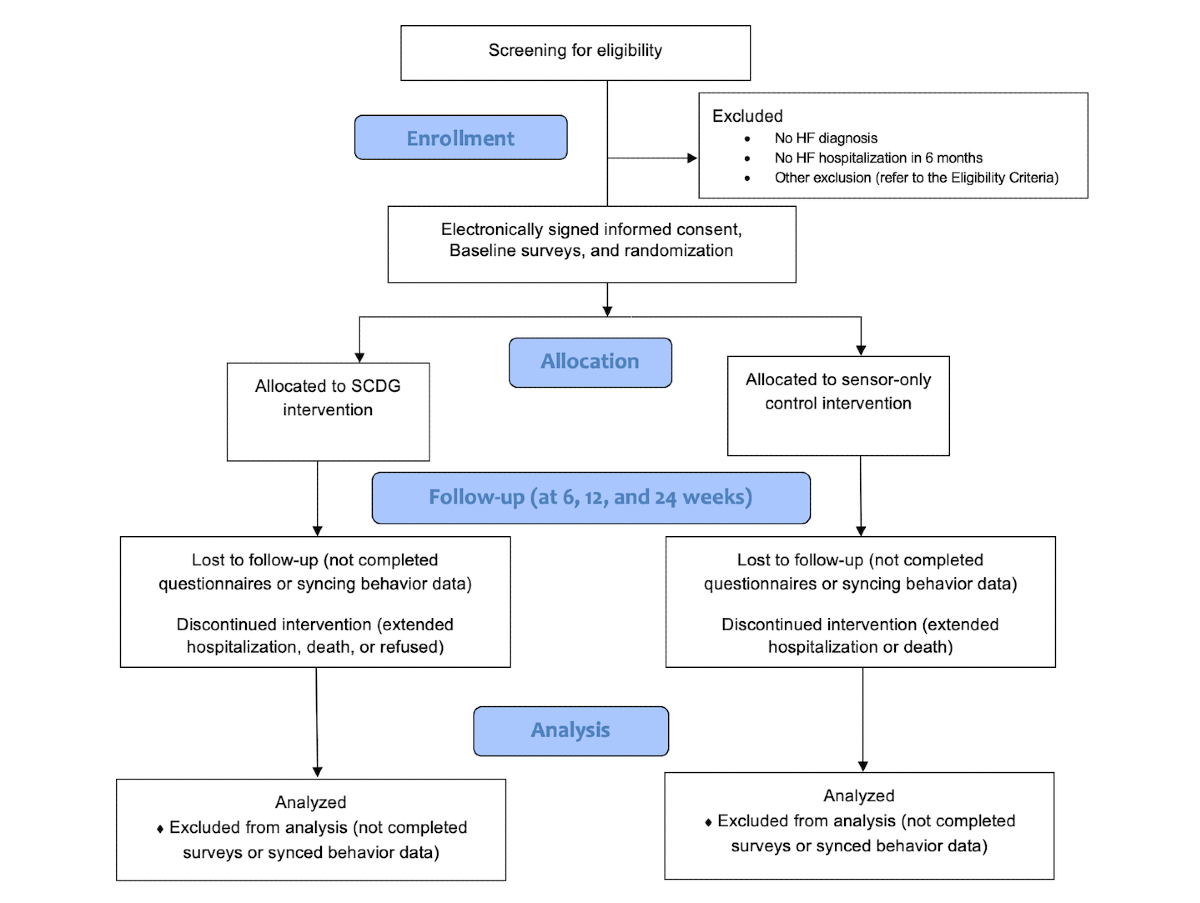
Study flow diagram. HF: heart failure; SCDG: sensor-controlled digital game.

An overview of the study’s phases is mapped in Figure 2.

### Participant Data Protection

We will take measures to ensure the best possible protection of participant data. Each participant will receive a unique identifier as well as a pseudonym identifier to use for their Withings account so that no personal or identifying information is connected to the account. The option to share contacts, location data, or photos from the game and the Withings app will be disabled. Withings is also subject to the European Union General Data Protection Regulation, which include strict policies against sharing of data or other misuse of data without participant knowledge or consent. Participants’ unique identifiers, rather than their personal information, will be connected to their behavioral and survey data as well as any data shared or presented externally. To protect participants’ privacy, a 6-digit unique identification number and dummy email addresses will be generated for the profile information required by the HealthMate app.

*Baseline demographic and clinical data* will include sex (male, female, or other), race and ethnicity, marital status, age, level of education, medications, depressive symptoms, hospitalizations, and HF-relevant phenotype data such as ejection fraction and comorbidities.

### Outcomes Measured

The outcome measures that we will use in the study are delineated and described in the following subsections.

#### Primary Outcome

The primary outcome assessed will be the *number of days reporting the HF self-management behavior of weight monitoring*: The data collection time frame will be 6, 12, and 24 weeks. This outcome will be measured by collecting the number of days with weight-monitoring data. These measures will be collected from the sensor logs within the Withings server from the HealthMate app [44] for the IG and CG.

#### Secondary Outcomes

We will assess a variety of secondary outcomes in the study. These outcomes are listed below.

1. *Daily physical activity steps*: The data collection time frame will be 6, 12, and 24 weeks. This outcome will be measured by collecting the number of daily cumulative steps days. Daily average total will be calculated weekly for 6, 12, and 24 weeks. These measures will be collected from the sensor logs within the Health Mate app [44] for the IG and CG.
2. *Daily physical activity intensity*: The data collection time frame will be 6, 12, and 24 weeks. This outcome will be measured by collecting the intensity level of daily physical activity. Averages will be calculated weekly for 6, 12, and 24 weeks. These measures will be collected from the sensor logs within the Health Mate app [44] for the IG and CG.
3. *Self-report HF self-management behaviors*: The data collection time frame will be baseline and 6, 12, and 24 weeks. This instrument will be the 9-item European Heart Failure Self-care Behavior Scale, which gives a standardized score from 0 to 100 (every item is given an equal weight), with a higher score indicating better self-care. Items are rated on a Likert scale ranging from 1 to 5. For calculating the standardized score, each item is reverse coded and then computed using the following formula: *(sum of all ever-coded items − 9) × 2.7777*. Cronbach α was .80 for the European Heart Failure Self-care Behavior Scale-9 and showed convergent validity with routine self-care behaviors and symptom response behaviors in the Self-Care of Heart Failure Index instrument [45].
4. *HF-related QoL*: The data collection time frame will be baseline and 6, 12, and 24 weeks. The instrument used will be items 13 to 15 from the Kansas City Cardiomyopathy Questionnaire (KCCQ). Values for the QoL domain range from 0 to 100, with higher scores indicating better QoL. The domain score will be transformed to a 0 to 100 range by subtracting the lowest possible scale score, dividing by the range of the scale, and multiplying by 100. Cronbach α was .78. The KCCQ is sensitive to clinical changes in HF compared with similar QoL instruments and had a significant, high correlation with the New York Heart Association HF classification (*P*<.001).
5. *HF-related functional status*: The data collection time frame will be baseline and 6, 12, and 24 weeks. Items 1 to 12 on the KCCQ will be used. Values for the functional status summary score ranged from 0 to 100, with higher scores indicating a better QoL. Summary score will be transformed to a 0 to 100 range by subtracting the lowest possible scale score, dividing by the range of the scale, and multiplying by 100. Cronbach α of the KCCQ for measuring functional status was excellent at .93. The scale demonstrated criterion validity with a high correlation with the New York Heart Association HF classification and the 6-minute walk test [46].
6. *Number of cardiac hospitalizations*: The data collection time frame will be baseline and 6, 12, and 24 weeks. This measure will be obtained through participant self-report through REDCap surveys and will be confirmed by hospitalization discharge summary and health care provider.
7. *HF self-management knowledge*: The data collection time frame will be baseline and 6, 12, and 24 weeks. The instrument used will be the 30-item Atlanta Heart Failure Knowledge Test [41]. Each correct answer is scored as 1 point, with no additional weighting of items; correct responses are then summed. Incorrect or skipped questions are awarded 0 points. Total scores range from 0 to 30. Higher scores indicate better knowledge of HF. Content validity ratings on relevance and clarity ranged from 0.55 to 1.0, with 81% of the items rated from 0.88 to 1.0. Reliability by Cronbach α was .84 for patients. Construct validity was demonstrated by directly correlating knowledge with clinical and self-care outcomes including dietary sodium consumption, medication adherence, and health care use.
8. *HF self-efficacy*: The data collection time frame will be baseline and 6, 12, and 24 weeks. The instrument used will be the 6-item Section C Self-efficacy section of the Self-Care of Heart Failure Index [42]. Scores on the self-efficacy scale will range from 0 to 100, with higher scores reflecting better self-efficacy. To standardize scores, the following formula will be used: *(sum of Section C items − 6) × 5.56*. Cronbach α was .88, with good evidence of construct validity and contrasting group validity.

To assess the facilitators and barriers impacting engagement of IG and CG participants with the SCDG and sensor-only interventions for HF self-management behavior adherence, we will use the convergence triangulation mixed methods design (including both quantitative and qualitative components) [48]. We will collect a survey informed by the Intrinsic Motivation Inventory [43] from all 200 participants at the 12-week follow-up assessment. In parallel, we will use random sampling to invite a subset (n=40) of IG and CG participants (20 per group) to participate in qualitative phone interviews at the end of 12 weeks. Interviews will be conducted by trained study staff, audio taped, and thematically coded by participant number to protect confidentiality. Participants will receive US $10 as an incentive to participate in the qualitative interview.

### Adverse Event Collection and Handling

Participants will be monitored for anticipated and unanticipated adverse events from the time they sign the study consent until 30 days after the final study visit. Participants will be provided with contact information of a research team member to inform them of any adverse events that occur. In addition, the study team will monitor the adverse events that arise during data collection. A communication protocol was developed to report adverse events to appropriate research personnel, including the development of a phone tree. Serious adverse events will be reported to the Institutional Review Board associated with the University of Texas Austin within 24 hours of being informed of the incident. Unanticipated problems will be reported by the principal investigator (KR) to the Data Safety Monitoring Committee, the funding agency, and the Institutional Review Board within 15 days of the event. The Data Safety Monitoring Committee will meet the principal investigator and study staff to review adverse events once a year and as needed to address any adverse events that need to be addressed immediately.

Should any conditions present harm to the participant if they continue with the intervention, they will either be withdrawn from the intervention or asked to take a break from the intervention until the condition subsides. These conditions will be assessed on a case-by-case basis, with the principal investigator (KR) making the final decision. All adverse events, protocol deviations, and reportable information will be entered into the REDCap database.

### Statistical Analysis

We have estimated the sample size for the primary outcome in our feasibility study for the 12 weeks study duration. All secondary outcomes will be examined on an exploratory basis. Analyses will be performed in an intent-to-treat manner.

### General Considerations

Cronbach α coefficients will be computed for each multi-item scale, with Cronbach α≥.70 denoting acceptable consistency. In outcome analyses, we will control for the possible effects of the biological variables of age and sex, prespecified as potentially strong predictors of outcomes. Statistical significance will be Cronbach α*=*.05 for primary and secondary analyses. Descriptive statistics will be computed for study variables to assess missingness, out-of-range values, and features of variable distributions including floor and ceiling effects. For testing and quantification of treatment effect with respect to primary and secondary outcomes, the IG and CG will be compared following the intention-to-treat principle.

### Missing Data

We expect missingness of items and other data to be minimal at baseline. Follow-up measures may be missing either because of formal dropout or failure to respond to contact from our research team. In all cases, we will assume missingness at random. For missing values at baseline, single (when <5%) or multiple (when ≥5%) imputation will be used. For more extensive missing data, especially at follow-up, we will use multiple imputation strategies when the analyses are based on generalized estimating equations [49] with sensitivity analyses to determine the effects of imputation. Sensitivity analyses will focus on which variables are used in imputation and on deviations from the missingness at random assumption (which must be parameterized, as this statistical assumption is not formally testable with the data) [50]. For missing outcomes in linear mixed models, imputation is not required because the analysis is based on maximum likelihood. However, missing predictors in mixed models will require single or multiple imputation.

### Measuring Outcomes

For the primary outcome of days with weight monitoring, binary weight-monitoring data (yes or no) will be summed over days for each of the 24 weeks during which data are collected. Data will be analyzed with a linear model for binomial-like data with an effect of time (ranging from 1 to 24) and a time-by-treatment effect. We do not need a treatment main effect owing to randomization, which implies no treatment effect at time=0. Primary outcome treatment effect at 12 weeks can then be estimated from the model as a difference in mean weight-monitoring days (out of 7) comparing the IG with the CG. The model will be fitted with generalized estimating equations [51], using the matrix-adjusted estimating equations method to estimate model correlation over repeated measures within a subject and using a bias-corrected sandwich variance estimator [52]. The model will allow for overdispersion relative to the binomial variance, yielding correct statistical descriptions of the data and inference. We will consider exchangeable, exponential decay and a combination of the 2 correlation structures. Finally, (1) we mention that the linear (vs the logistic) model is valid for binomial data, especially when the binomial parameter is not near 0 or 1 and yields an interpretable treatment effect on the scale of “number of weight-monitoring days,” and (2) we will maximize power by analyzing all 24 weeks of data, although we aim to extract the 12-week mean difference as the primary contrast of interest.

For the secondary outcome of physical activity, as measured by the number of steps, data will again be aggregated (summed) by week. As the measures are counts, we will use a log link and variance proportional to the mean (as with the Poisson distribution), allowing for an overdispersion parameter. Analysis will then emulate that for the number of weight-monitoring days. The log link will yield coefficients that, when exponentiated, will be interpreted as relative mean numbers of steps, which, given the expected between-person variability, will be more interpretable than a mean difference on the natural scale. For secondary outcome measures of HF self-management knowledge, self-efficacy, HF-related functional status, and QoL measured 4 times over the multiple data collection time points, we will use linear mixed effects models [53] with random slopes and intercepts to capture the association over time in the repeated measures within participant, estimating the treatment effect from the model at each of 6, 12, and 24 weeks.

### Sample Size and Statistical Power

For a 1:1 randomization scheme (IG:CG), we aim to recruit 100 participants per group and allow for 25% attrition (some of which will be recovered through the treatment of missing data). We would like to detect a weight-monitoring rate of at least 80% (5.6/7) of days per week, as patients with HF who completed ≥80% of weight diaries were found to have significantly reduced odds for HF-related hospitalizations compared with patients who completed <80% of weight diaries [7]. In our planned analysis, we aim to use weekly data for weeks 1 through 24, fitting a flexible (eg, *df*=2) trend for the mean number of days (out of 7) in which participants complete weight monitoring, and thereby extracting the estimate at 12 weeks for each of the IG and CG. Using all 24 weeks of data represents an efficient use of data by exploiting a smooth time trend, by leveraging the positive correlation among the repeated measures on a given individual, and by offering the opportunity to handle missing time points through imputation or weighting of the nonmissing longitudinal data. For power estimation, because we are not sure about the form of the model based only on pilot data and because pilot data are relatively sparse for purposes of estimating within-subject correlation, we *conservatively* (in the sense that true power is expected to be greater) use aggregated pilot data just across weeks 7 to 12 (42 days) to estimate the mean and variance of *â*, where *a* is the average within-group probability of engaging in weight monitoring on any given day. Owing to the strong day-to-day correlation within subject, the total number *Y* of weight-monitoring days is considerably overdispersed relative to the binomial distribution, such that for a given subject in a given group, the mean of *Y* is 42*a* and the variance of *Y* is 42*Øa*(1 – *a*), where *Ø* is an overdispersion parameter. In our pilot data, we estimated *Ø*=17.5; these values yield 2-group power calculations comparing IG weight-monitoring rate, *a_IG_*, with CG weight-monitoring rate, a_CG_, under within-group sample size n=75. If *a_IG_*=0.80, then we will have 90% power to detect differences between *a_IG_* and *a_CG_*, as small as 0.14 (SD 0.12; ie, *a_CG_* as big as 0.66, SD 0.68), yielding strong power from our longitudinal design. Owing to the need to estimate the overdispersion parameter, these calculations were custom coded by our team in R (R Foundation for Statistical Computing) [54].

### Qualitative Coding and Analysis

Survey analysis will include descriptive statistics of counts and frequencies for the scale items and thematic analysis for the open-ended survey responses [55,56]. Verbatim transcripts will be created; they will be coded and examined using thematic analysis and Dedoose (SocioCultural Research Consultants) [57] software to enable examination of themes overall. Finally, the quantitative and qualitative results will be merged [48] to provide insights on the weight monitoring, physical activity, sensor use, and SCDG-playing behaviors of participants with HF; facilitators and barriers affecting participant engagement with sensors or SCDG for HF self-management behavior adherence; as well as future improvements and design modifications of the SCDG. The facilitators and barriers affecting engagement in sensor-based digital health interventions will be published in a secondary analysis publication.

## Results

A CONSORT (Consolidated Standards of Reporting Trials) flow diagram will be reported. The clinical trial was initiated on October 24, 2022, and the first participant was enrolled on November 7, 2022. Recruitment of the last participant is anticipated in quarter 1 of 2025. Full trial results are planned to be published by 2026.

## Discussion

### Principal Findings

Our SCDG intervention addresses a substantial challenge, that is, to develop an affordable and scalable technology-assisted intervention to facilitate persistent adherence to self-management behaviors by adults with HF to improve QoL, functional status, and health outcomes and, ultimately, to reduce the burden of HF worldwide. We hypothesize that 12 weeks of playing the SCDG will result in greater behavioral changes in key self-management indicators of HF outcomes in adults aged >45 years compared with a CG that uses only sensors.

The recent rapid rise in the use of smartphones across demographic groups has enabled the scalability of innovative digital health interventions to promote HF self-management behaviors across diverse groups [58]. Although interventions such as digital games for health can be easily deployed to address geographic disparities in HF outcomes experienced by populations in southern US states, there is a lack of systematic research on the effectiveness of digital game interventions to promote evidence-based self-management behaviors of individuals with HF with conflicting priorities or in unsupportive environments. Current digital health interventions with unobtrusive monitoring capabilities allow the gathering of relevant contextual data to tailor and adapt interventions without overburdening participants through cumbersome data collection [59]. This study protocol was informed by our feasibility randomized controlled trial study, in which we demonstrated the remote implementation of a smartphone-based intervention using a digital game and sensors among participants in 18 counties in Texas and Oklahoma [29]. Combining digital games and sensors holds the potential to offer a powerful way to improve treatment adherence [60,61]; sustain healthy behaviors; and make health care more participatory, personalized, predictive, and preventive, as defined by precision medicine [62]. The lessons from this initial study provide a strong foundation to test our intervention with individuals with HF in 7 southern US states. Therefore, we hypothesize that 12 weeks of playing the SCDG will result in greater behavioral changes in key self-management indicators of HF outcomes in adults aged >45 years compared with a CG that uses only the sensors.

### Strengths

Developing a dashboard that will allow easier visualization of behavioral data from all participants will provide us with the opportunity to intervene immediately in the event of absent or unsafe levels of behavioral data. In addition, fully remote study procedures will enhance the accessibility of HF research to participants living in rural or remote areas that are far from large HF centers in cities.

### Limitations

Self-selection bias may be a limitation of this study as participation is voluntary, and participants already comfortable with using technology might be especially motivated to participate in digital intervention studies.

### Dissemination Plan

We will disseminate our study findings to target an audience that cares for patients with HF and will benefit from the lessons learned through our study. This includes disseminating our findings through (1) peer-reviewed health technology–related journals such as *Games for Health Journal* or *Journal of Medical Internet Research* and clinical journals such as *Journal of Cardiovascular Nursing* or *Journal of Cardiac Failure*; (2) annual research conferences conducted by the Heart Failure Society of America, American Heart Association, Society of Behavioral Medicine, American Medical Informatics Association, and Gerontological Society of America; (3) local and regional nursing organizations such as Texas Public Health Association’s monthly webinars and similar organizations in other southern US states; (4) scheduled continuing education sessions or monthly meetings conducted by HF clinics for their staff; and (5) local and regional support groups for patients with HF. In addition, all peer-reviewed and accepted manuscripts will be uploaded either by the investigator or by the journal (for those journals that perform this function) to the National Library of Medicine’s PubMed Central in accordance with the National Institutes of Health Public Access Policy.

### Conclusions

Nevertheless, this project will generate insight and guidance for scalable and easy-to-use digital gaming solutions to motivate persistent adherence to HF self-management behaviors and improve health outcomes among individuals with HF.

## Appendix

Multimedia Appendix 1 Peer review report.

## Abbreviations

CG: control group
CONSORT: Consolidated Standards of Reporting Trials
HF: heart failure
IG: intervention group
KCCQ: Kansas City Cardiomyopathy Questionnaire
QoL: quality of life
REDCap: Research Electronic Data Capture
SCDG: sensor-controlled digital game

## Data Availability

Although the final data set comprising questionnaire and qualitative data will be stripped of identifiers, we believe that there remains the possibility of deductive disclosure of participants with unusual characteristics. Thus, we will make the data and associated documentation available to users only under a data-sharing agreement that provides for (1) a commitment to using the data only for research purposes and not to identify any individual participant, (2) a commitment to securing the data using appropriate computer technology, and (3) a commitment to destroying or returning the data after analyses are completed. After reviewing the purpose of the data request to ensure that it is consistent with the original project goals and verifying that the request meets approval from the Institutional Review Board, deidentified data may be shared with researchers as soon as possible but no later than within 1 year from the completion of the funded project period for the parent award or upon acceptance of the data for publication, whichever is earlier.
