# Supplementary material for: Sensor-Controlled Digital Game for Heart Failure Self-management: Protocol for a Randomized Controlled Trial
Source: JMIR Res Protoc. 2023 May 10;12:e45801. doi: 10.2196/45801 (PMC10209796; doi:10.2196/45801)
Supplement: Multimedia Appendix 1 [file resprot_v12i1e45801_app1.pdf]

SUMMARY STATEMENT
(Privileged Communication)

PROGRAM CONTACT:
ERIN ITURRIAGA
301-435-0403
iturriae@mail.nih.gov

Release Date: 06/23/2021
Revised Date:

Application Number: 1 R01 HL160692-01

Principal Investigator

RADHAKRISHNAN, KAVITA

Applicant Organization: UNIVERSITY OF TEXAS, AUSTIN

Review Group: BMHO
Biobehavioral Medicine and Health Outcomes Study Section

Meeting Date: 06/07/2021
Council: OCT 2021
Requested Start: 09/01/2021
RFA/PA: PA18-722
PCC: HHATMN
Dual IC(s): NR, EB

Project Title: Sensor-controlled digital game for heart failure self-management behavior adherence: A randomized controlled trial
SRG Action: Impact Score:35 Percentile:24
Next Steps: Visit https://grants.nih.gov/grants/next_steps.htm
Human Subjects: 48-At time of award, restrictions will apply
Animal Subjects: 10-No live vertebrate animals involved for competing appl.
Gender: 1A-Both genders, scientifically acceptable
Minority: 1A-Minorities and non-minorities, scientifically acceptable
Age: 3A-No children included, scientifically acceptable

Project Year	Direct Costs Requested	Estimated Total Cost
1	415,447	639,208
2	479,889	738,358
3	482,293	742,057
4	432,195	664,976
TOTAL	1,809,824	2,784,599

ADMINISTRATIVE BUDGET NOTE: The budget shown is the requested budget and has not been adjusted to reflect any recommendations made by reviewers. If an award is planned, the costs will be calculated by Institute grants management staff based on the recommendations outlined below in the COMMITTEE BUDGET RECOMMENDATIONS section.
EARLY STAGE INVESTIGATOR
NEW INVESTIGATOR

1R01HL160692-01 Radhakrishnan, Kavita

COMMITTEE BUDGET RECOMMENDATIONS
EARLY STAGE INVESTIGATOR
NEW INVESTIGATOR
PROTECTION OF HUMAN SUBJECTS UNACCEPTABLE

RESUME AND SUMMARY OF DISCUSSION: This application proposes a two-armed randomized controlled trial (RCT) to refine and test efficacy of a smartphone sensor-controlled digital games (SCDGs) intervention to improve self-management of behaviors including weight monitoring (WM) and physical activity (PA) among adults with heart failure (HF). During discussion, the panel agreed the significance of a remote, scalable intervention to improve self-management behaviors in HF patients is high, with potential to address geographical and racial health disparities in HF outcomes, reduce rehospitalizations and increase quality of life. Reviewers noted the use of a remote platform with SCDG that includes integrated real-time sensor monitored health behaviors to promote adherence to self-management behaviors by this promising new investigator and her strong investigative team is innovative. Additional strengths include pilot data supporting feasibility and acceptability of SCDGs by older adults, a recruitment plan targeting states with highest prevalence of and health disparities in HF and a rigorous design with an active control group, appropriate measures and qualitative assessment of participant perspectives to improve the intervention. The panel identified several weaknesses including underdeveloped rationale for WM and details of PA analyses, generalizability limitations due to use of a recruitment company and the ecological momentary analysis approach was not well-articulated. Overall, the panel agreed the application's strengths outweighed its weaknesses and will have a moderately high impact on addressing geographical and health disparities, as well as reduce rehospitalizations in heart failure patients.

DESCRIPTION (provided by applicant): Heart failure (HF) is a growing pandemic; in the U.S., the number of those diagnosed with HF is expected to rise to 8 million with annual costs at $69 billion by 2030. Despite effective interventions to reduce HF morbidity and mortality, vulnerable populations with HF suffer disproportionately from hospitalization and mortality especially in the southern U.S. states. Self-management (SM) behavioral interventions to improve HF outcomes are therefore imperative. Remote interventions to promote SM behaviors present an important strategy to address the widening geographical and racial health disparities in HF outcomes. One promising approach is the use of sensor-controlled digital games (SCDGs), which offer affordable, portable, scalable tools to facilitate engagement in HF SM behaviors that show the poorest adherence (weight monitoring and physical activity) while being enjoyable and easy to use. The primary goal of this study is to evaluate the efficacy of a SCDG intervention that integrates HF participants' behavioral data from weight scale and activity tracker sensors to activate game progress, rewards, and feedback. For Aim 1, we will refine an SCDG that we have already developed for mobile smartphones to be playable for longer durations for sustained behavior adherence to weight-monitoring and physical activity. For Aim 2, using a randomized controlled clinical trial, we will compare the SCDG intervention versus a sensor-only intervention for the primary outcome of rate of engagement in the HF SM behavior of weight-monitoring and the secondary outcomes of physical activity behavior engagement, HF SM knowledge, self-efficacy, HF functional status, hospitalization, and quality of life at baseline and at 6, 12, and 24 weeks. For our sample, we will recruit adults aged 45 years or older from 7 southern U.S. states and hospitalized with HF within the past 6 months. We will randomize 200 participants to either the SCDG intervention group, in which participants will receive sensors that track weight monitoring and activity and will play the SCDG on a mobile smartphone, or a control group that will receive sensors, an app that tracks activity and weight monitoring, and standardized written HF educational materials. For Aim 3, we will conduct a mixed-methods assessment to discern facilitators and barriers impacting

participants' engagement with the sensor-based interventions for HF SM behavior adherence. For Aim 4, based on daily HF SM weight-monitoring and physical activity behavior data and ecological momentary assessments of symptoms, mood, satisfaction, and cognitive status, we will conduct digital phenotyping of HF SM weight- monitoring and physical activity behaviors. This project will generate insight and guidance for scalable and easy-to-use digital gaming solutions to motivate persistent adherence to HF SM behaviors and improve health outcomes among individuals with HF.

PUBLIC HEALTH RELEVANCE: Individuals diagnosed with heart failure (HF) in the southern U.S. face significant disparities in hospitalization and mortality rates, but effective HF self-management behaviors can reduce their healthcare burden and suffering. We propose a randomized controlled trial of a sensor-controlled digital game intervention in 7 southern U.S. states, to improve HF individuals' knowledge, skills, and motivation to engage in the important HF self-management behaviors of weight monitoring and physical activity. This project will generate insight and guidance regarding the use of digital gaming as an affordable, portable, scalable technology to improve HF self-management behaviors and potentially reduce the burden of HF.

CRITIQUE 1

Significance: 3
Investigator(s): 3
Innovation: 2
Approach: 4
Environment: 2

Overall Impact: This study addresses an important topic in that HF is a major contributor to hospitalizations, adverse prognosis, and health care costs. There is poor adherence to HF self-management behaviors that can improve outcomes, and an ongoing need for scalable interventions that work. The investigator team, led by a promising ESI, is strong, with a track-record of collaborating on a pilot feasibility study, and complementary expertise. There are several innovative aspects, most notably, designing a sensor-controlled digital game (SCDG) for predominantly older adults with HF is an innovative application of digital game design. The integration of real-time sensor monitored health behaviors allows for monitoring and feedback, potentially powerful behavior change technique and will also allow for predictive modeling of health behaviors – though this latter goal is less novel. There are several strengths of the approach, including the use of a randomized design to test the efficacy of the intervention; enrollment from 7 US southern states with the highest prevalence of HF; pilot data demonstrating at least some enthusiasm and acceptability of DGs in older adults with HF as well as ability to recruit patients remotely with decent engagement in the SCDG and end-of-study qualitative assessments. Enthusiasm was diminished for several reasons. It was not clear why weight monitoring (WM) was selected as the primary outcome given less clear linkage of WM with improved prognosis compared to other behaviors. Few details were provided as to how physical activity (PA) analyses will be conducted, including whether they will assess intensity of activity versus step counts, alone. The statistical power for the PA analyses was not described. WM is only helpful if linked with adherence and titration of diuretic medications and low salt diet – yet intervening on these behaviors is described as a future direction. The recruitment approach through a clinical trial recruitment company may limit the understanding of the generalizability of the intervention. Feasibility of EMA assessments for Aim 4 was unclear as EMA was not included in the pilot study, and there was little mention of statistical power for this Aim. The environment was satisfactory for the proposed research. Overall, this study was expected to have moderate potential for impact, driven primarily by its thoughtful design, innovation, and potential

to more broadly inform the understanding of the potential for gaming interventions to impact adherence in older adults with chronic disease, while tempered by its incremental approach, with the trial focused on weight monitoring and PA rather than the full set of behaviors relevant to HF outcomes.

1. Significance:

Strengths

- HF is a substantial contributor to adverse health outcomes and costs
- There is a need for scalable, potent interventions that improve HF self-management behaviors including PA and weight monitoring.
- Digital phenotyping HF behaviors, while exploratory, may inform design of sensor-controlled behavioral interventions

Weaknesses

- No major weaknesses

2. Investigator(s):

Strengths

- Led by an ESI with background in engineering and nursing, conducted R21 funded pilot feasibility trial
- Well supported by experts in computer science, gaming design, mobile health sensors, biostatistics

Weaknesses

- Unclear extent of experience on team in game design for older adults and in conduct of behavioral RCTs
- HF specialists have small role on team as consultants
- Unclear expertise in actigraphy assessment of physical activity

3. Innovation:

Strengths

- Applying SCDG to older adults with HF is innovative; has potential to broadly inform the utility of this approach for older adults, at least in terms of engagement

Weaknesses

- Physical activity monitoring focused on step counts – does not measure sedentary behavior or exercise intensity or sleep as could be measured with actigraphy

4. Approach:

Strengths

- Builds on pilot data from feasibility trial which demonstrated decent engagement with prototype for the SCDG
- Uses a rigorous randomized design to study efficacy

- Enrolls patients remotely across 7 states with high prevalence of HF
- Includes rigorous qualitative assessment of participants perspectives on the intervention at end of study which should inform subsequent intervention development and readiness for implementation
- Explicitly considers sex as a biological variable even stratifying randomization by sex

Weaknesses

- Approach does not target medication and dietary adherence which are key to HF outcomes, and is not integrated with care pathways, likely influencing potency
- Few details on how PA will be calculated, nor on statistical analyses involving PA
- The Withings Move activity tracker can calculate intensity of exercise and sleep, but only step counts are mentioned in the approach
- Relies on patient self-report to identify potential HF hospitalizations; few details on how hospitalizations will be adjudicated
- Functionality of Withings apps for control group not clearly described – there are some sensor-controlled gaming aspects of these apps making it a little challenging to understand the key difference between intervention and control groups.

5. Environment:

Strengths

- Access to computer sense and clinical trial infrastructure through institution

Weaknesses

- No major weaknesses

Study Timeline:

Strengths

- Clearly described and realistic milestones

Weaknesses

- No major weaknesses

Protections for Human Subjects:

Acceptable Risks and/or Adequate Protections

Data and Safety Monitoring Plan (Applicable for Clinical Trials Only):

Acceptable

Inclusion Plans:

- Sex/Gender: Distribution justified scientifically
- Race/Ethnicity: Distribution justified scientifically
- Inclusion/Exclusion Based on Age: Distribution justified scientifically

- Enrollment expected to have substantial diversity including 20% AA and 10% Hispanic, 50% women

Vertebrate Animals:

Not Applicable (No Vertebrate Animals)

Biohazards:

Not Applicable (No Biohazards)

Resource Sharing Plans:

Acceptable

Budget and Period of Support:

Recommend as Requested

CRITIQUE 2

Significance: 4
Investigator(s): 4
Innovation: 3
Approach: 5
Environment: 2

Overall Impact: This is a proposal to utilize a rigorous RCT design to test an innovative self-care and gamification app against an active control group (that receives all but the 'app'), with the primary outcome being an essential component of heart failure (HF) self-care. The aim to recruit previously hospitalized HF patients from underserved parts of the US (e.g., high risk for re-hospitalization) using a completely remote research platform, contribute to the significance of the undertaking because of the potential disseminability. This is a strong research team that together has accomplished the R21 work that informs the proposal, though overall, the experience managing an R01 based RCT of this size resides in consultant Baranowski, and involvement of cardiologist consultants does not inform the undertaking. This work demonstrates scientific rigor, premise, and feasibility. Scientific rigor is also demonstrated in the approach to measurement, which relies on well-validated instruments. The use of an active control group provides an additional element - e.g., if both groups reach/improve to a clinically meaningful level of daily weighing, then it will indicate that the larger platform may not be necessary, though the proposed secondary analyses (e.g., those associated with SA 3 & 4) will help identify which patients would benefit sufficiently from each approach. While enthusiasm is high, some elements are not fully described. It appears that some degree of EMA will be incorporated to identify factors associated with adherence/use of the app vs. non-adherence/not using the app, and this component is unclear. The pilot data provided describe percent change, and 1) group differences are not apparent, and 2) achievement of threshold adherence linked to outcomes - e.g., 80% daily weighing, increase of 2,000 steps per day up to 10,000 daily steps, is not described. In addition, how physical activity goals will be established is not clear. The environment is strong.

1. Significance:

Strengths

- Focus on (non) adherence in heart failure (HF) among patients with prior HF hospitalization is significance, as this is a group at risk for repeat hospitalization and health decline.
- Targeting daily weighing as primary outcome as a proximal factor that presages decompensation
- Testing a 'gamification' platform with 'app' and both weight and physical activity sensors enhances disseminability, should the intervention have demonstrated efficacy over the control condition.
- Use of a remote platform for all research activities, which allows for recruitment over a wide swath of the US having underrepresentation of healthcare and poorer HF related outcomes.
- Even if there are no group differences at follow-up, but both groups show improved adherence, there will be an approach to disseminate.

Weaknesses

- Key preliminary data that support rigor and premise are not sufficiently informative - e.g., they applicant describes an increase in weighing and physical activity, but for weight, this is not 'tagged' to the 80% threshold powered to in the current application, and the increase of 623 steps per day is only 31% of the 2000 that is associated with meaningful outcomes.

2. Investigator(s):

Strengths

- This is a very strong team that brings expertise in all elements of this moderately complex undertaking
- The team has collaborated previously in accomplishing the work that informs the current application.

Weaknesses

- Aside from consultant Baranowski, this would be the first NIH funded R01 of an RCT, which as described is a considerable undertaking
- There are 3 consultant cardiologists who provide apparent overlapping expertise. Furthermore, the importance of this expertise for the proposed RCT is not fully apparent, as the PI has experience in HF care and the patients will all be recruited and engaged remotely - e.g., these cardiologists likely are not involved in the care of these patients.

3. Innovation:

Strengths

- Integration of real time data with a gamification 'app' to promote adherence to key HF self-management behaviors.
- Utilization of EMA to ascertain intra-personal experiences associated with adherence/use of the 'app' with non-adherence/not using the 'app'
- Breath of data to be collected, including variables associated with adherence/app use and non/adherence/non app use provide for personalization through phenotyping

- Use of a remote platform for all research activities, which allows for recruitment over a wide swath of the US having underrepresentation of healthcare and poorer HF related outcomes.

Weaknesses

- Dissemination of new technologies that monitor a more proximal index of ‘fluid load’ that leads to weight gain - e.g., implanted pulmonary artery pressure monitors, may make the issue of daily weighing less important.
- Behaviors that contribute to weight gain in HF - e.g., dietary and medication non-adherence, might provide better targets.

4. Approach:

Strengths

- Use of highly rigorous RCT design
- Measures to be used are all validated with this population, thus demonstrating scientific rigor.
- Prior work supports scientific premise and feasibility, including ability to recruit, ability to deliver the intervention and control conditions, and high satisfaction/uptake of the platform.
- Use of a remote platform for all research activities, which allows for recruitment over a wide swath of the US having underrepresentation of healthcare and poorer HF related outcomes.
- Combined SA 3 and SA 4 should provide for greater personalization and implementation testing
- Use of an active control arm a potential strength - e.g., it may not be necessary to utilize the whole gamification element to achieve clinically meaningful level of adherence to HF self-care, though here too, apparent secondary analyses - e.g., those associated with SA 4 - can help identify those patients for whom this extra element are required to achieve sufficient self-management.

Weaknesses

- EMA platform/questions are not fully described. It is not apparent whether this will be incorporated into the ‘app’ or will be a distinct platform. Impression is that this is underdeveloped
- Data from the pilot study mostly describe percent increase (e.g., in adherence), rather than the more informative baseline and follow-up values, or the percent in each group that met some threshold (e.g., 80% adherence to daily weight monitoring, average 2,000 step increase in PA per day).
- Pilot data (Figure 3) don’t appear to show real group differences.
- Increasing dissemination of more proximal indices of weight gain - e.g., pulmonary artery pressure monitoring - may make focus on daily weighing less relevant in the near term.
- The description of a key game element is helping the avatar avoid hospitalization. Given that this is a relatively high-risk group (e.g., prior HF hospitalization), this outcome could happen despite high adherence to PA and daily weighing, setting up a disconnect between being a good game player and a real-world outcome.
- Previously, updates to hardware/software became an implementation problem. Given reliance on commercial products, this might be a continued problem of interface with the ‘app’.
- The manner by which initial goals for PA - or for increases over the weeks of participation - will be set, are not apparent.

5. Environment:

Strengths

- UT Austin, including the Advanced Computer Center and School of Engineering - provides a very strong research environment

Weaknesses

- None noted

Study Timeline:

Strengths

- A detailed graphic timeline with appropriate milestones is provided

Weaknesses

- It is not clear how much time will be needed to refine the SCDG intervention. 6-months is designated in the timeline, but the Research Plan describes a 12-month period.

Protections for Human Subjects:

Unacceptable Risks and/or Inadequate Protections

- The applicant describes risks associated with potential loss of confidentiality and protections against this risk. Yet, the establishment of PA at initiation of trial participation, and of activity goals moving forward, and the potential risks associated, are not discussed.
- Furthermore, it is possible that an adherent patient who uses the app daily could still have a HF hospitalization, 'setting up a disconnect' between what the 'app' (working through the avatar) is promoting, and the lived experience.

Data and Safety Monitoring Plan (Applicable for Clinical Trials Only):

Acceptable

- Applicant describes formation of a DSMB, and appropriately frequent monitoring of adverse events.

Inclusion Plans:

- Sex/Gender: Distribution justified scientifically
- Race/Ethnicity: Distribution justified scientifically
- Inclusion/Exclusion Based on Age: Distribution justified scientifically
- Plan is to recruit equal numbers of men and women, 66% White. No upper age limit to participation. Age> 45 is appropriate, given the nature of the population - previously hospitalized HF.

Vertebrate Animals:

Not Applicable (No Vertebrate Animals)

Biohazards:

Not Applicable (No Biohazards)

Resource Sharing Plans:

Acceptable

Budget and Period of Support:

Budget Modifications Recommended (in amount/time)

Recommended budget modifications or possible overlap identified:

- The need to involve the multiple cardiologist consultants who each provide the same expertise is not apparent, given the experience in HF that the PI brings, and the fact that all patients will be recruited and involved remotely

CRITIQUE 3

Significance: 1
Investigator(s): 1
Innovation: 1
Approach: 2
Environment: 1

Overall Impact: HF is a morbid disease and the overall morbidity and mortality continue to increase, despite having many advanced interventions and therapies. Remote interventions to promote self-management (SM) behaviors present an important strategy to address the widening geographical and racial health disparities in HF outcomes. The team proposes that sensor-controlled digital games (SCDGs), is an affordable, portable, scalable tool to facilitate engagement in HF SM behaviors. Aim 1 will refine the SCDG, Aim 2 will be a RCT comparing SCDG vs sensor-only intervention, Aim 3 will be a mixed-methods assessment to discern facilitators and barriers impacting participant engagement, and Aim 4 will phenotype HF SM weight-monitoring and physical activity. I am very enthusiastic about this proposal. The proposal addresses a clinically significant problem in a very innovative way by using the SCDG. Data demonstrate feasibility, and rigor is demonstrated in the methods and approach. The team has great synergy and have an established history of collaborative grants and publications. UT Austin is a supportive academic environment with plenty of resources available to support the team.

1. Significance:

Strengths

- HF is a growing pandemic; in the U.S., the number of those diagnosed with HF is expected to rise to 8 million with annual costs at $69 billion by 2030.
- HF is the most common diagnosis among hospitalized patients aged 65 years or older, with a 50% death rate within 5 years following diagnosis
- SM interventions to improve HF outcomes are imperative, because the inability to self-manage HF results in frequent hospitalizations and poor QoL

Weaknesses

- None

2. Investigator(s):

Strengths

- PI has a background in engineering and informatics, ESI with a history of NIH-funding (R21).
- R21 led by PI led to the development of a sensor-controlled digital game (to be used in the current R01)
- Excellent team with a history of collaboration:
  - Mobile computing for real-time behavior data aggregation from sensors (Christine Julien)
- Real-time behavior data analysis using machine learning techniques (Edison Thomaz),
- Digital gaming for behavior change (Tom Baranowski and Matthew O'Hair),
- Delivery of community-based interventions to diverse racial groups (Miyong Kim)
- History of previous funding and publications
- Experienced statistician (Rathouz) with previous experience in patient behaviors
- HF cardiologists/consultants from 3 different systems

Weaknesses

- None

3. Innovation:

Strengths

- The SCDG intervention will provide contextually relevant feedback and game incentives to motivate behavioral changes in HF SM,
- The SCDG intervention will collect data on participants' responses to the feedback to inform decision-making
- In the feasibility RCT study, the team demonstrated remote implementation of a smartphone-based intervention using a DG and sensors among participants in 18 counties which included rural areas that were 100 miles away from the closest city in Texas and Oklahoma.

Weaknesses

- None

4. Approach:

Strengths

- Key preliminary data: randomized feasibility trial of SCDG intervention showed 46% increase in weighing 5 days or more in a week from baseline to 12 weeks and 23% increase in physical activity steps from 7th to 12th week as compared to the sensor-only control group.
- Feasibility study demonstrated ~90% acceptability of SCDG
- Rigor has been demonstrated by citing key data supporting SCDG, using a RCT design (computer automated), appropriate blinding of key personnel, and detailed methodological approach.

- Multiple team members participated in the SCDG feasibility 2-group RCT, and they have an established collaboration that developed the application programming interface required for the app.
- Key refinement modifications will be implemented, include synchronizing of data and improved cognitive assessments
- Statistical plan is rigorous and detailed

Weaknesses

- Preliminary data were not statistically significant with respect to the WM changes in the SCDG group

5. Environment:

Strengths

- UT Austin is an excellent research University/academic environment
- Excellent research and SON. Plenty of resources and support available to investigators including the Biomedical Data Science Hub and Center for Transdisciplinary Collaborative Research in the SON

Weaknesses

- None

Study Timeline:

Strengths

- Incredibly detailed, easy to follow, and appears to be feasible

Weaknesses

- None

Protections for Human Subjects:

Acceptable Risks and/or Adequate Protections

- No concerns

Data and Safety Monitoring Plan (Applicable for Clinical Trials Only):

Acceptable

- Minimal risk

Inclusion Plans:

- Sex/Gender: Distribution justified scientifically
- Race/Ethnicity: Distribution justified scientifically
- Inclusion/Exclusion Based on Age: Distribution justified scientifically
- Age of 45 is used, and evidence shows that lifetime risk for HF is highest for those >45 years of age

Vertebrate Animals:
Not Applicable (No Vertebrate Animals)

Biohazards:
Not Applicable (No Biohazards)

Resource Sharing Plans:
Not Applicable (No Relevant Resources)

Budget and Period of Support:
Recommend as Requested

THE FOLLOWING SECTIONS WERE PREPARED BY THE SCIENTIFIC REVIEW OFFICER TO SUMMARIZE THE OUTCOME OF DISCUSSIONS OF THE REVIEW COMMITTEE, OR REVIEWERS' WRITTEN CRITIQUES, ON THE FOLLOWING ISSUES:

PROTECTION OF HUMAN SUBJECTS: UNACCEPTABLE
During the discussion the panel agreed that any physical activity involved with the intervention increases potential risk and since no plan for addressing such risk is included in the application, the protection of human subjects is unacceptable.

INCLUSION OF WOMEN PLAN: ACCEPTABLE

INCLUSION OF MINORITIES PLAN: ACCEPTABLE

INCLUSION ACROSS THE LIFESPAN: ACCEPTABLE

COMMITTEE BUDGET RECOMMENDATIONS: The panel agreed that the need for multiple cardiologist consultants, given the PI's expertise, was not warranted.

Footnotes for 1 R01 HL160692-01; PI Name: Radhakrishnan, Kavita

NIH has modified its policy regarding the receipt of resubmissions (amended applications).See Guide Notice NOT-OD-18-197 at https://grants.nih.gov/grants/guide/notice-files/NOT-OD-18-197.html. The impact/priority score is calculated after discussion of an application by averaging the overall scores (1-9) given by all voting reviewers on the committee and multiplying by 10. The criterion scores are submitted prior to the meeting by the individual reviewers assigned to an application, and are not discussed specifically at the review meeting or calculated into the overall impact score. Some applications also receive a percentile ranking. For details on the review process, see http://grants.nih.gov/grants/peer_review_process.htm#scoring.

MEETING ROSTER

Biobehavioral Medicine and Health Outcomes Study Section
Risk, Prevention and Health Behavior Integrated Review Group
CENTER FOR SCIENTIFIC REVIEW
BMHO
06/07/2021 - 06/08/2021

Notice of NIH Policy to All Applicants: Meeting rosters are provided for information purposes only. Applicant investigators and institutional officials must not communicate directly with study section members about an application before or after the review. Failure to observe this policy will create a serious breach of integrity in the peer review process, and may lead to actions outlined in NOT-OD-14-073 at https://grants.nih.gov/grants/guide/notice-files/NOT-OD-14-073.html and NOT-OD-15-106 at https://grants.nih.gov/grants/guide/notice-files/NOT-OD-15-106.html, including removal of the application from immediate review.

CHAIRPERSON(S)

FILLINGIM, ROGER B, PHD
DISTINGUISHED PROFESSOR
DEPARTMENT OF COMMUNITY DENTISTRY
AND BEHAVIORAL SCIENCE
COLLEGE OF DENTISTRY
UNIVERSITY OF FLORIDA
GAINESVILLE, FL 32610

MEMBERS

BENJAMIN- NEELON, SARA E, PHD
PROFESSOR
DEPARTMENT OF HEALTH
BEHAVIOR AND SOCIETY
JOHNS HOPKINS SCHOOL OF PUBLIC HEALTH
BALTIMORE, MD 21205

BRUEHL, STEPHEN, PHD
PROFESSOR
DEPARTMENT OF ANESTHESIOLOGY
VANDERBILT UNIVERSITY MEDICAL CENTER
NASHVILLE, TN 37212

BUCK, HARLEAH G, PHD *
PROFESSOR
COLLEGE OF NURSING
UNIVERSITY OF IOWA
IOWA CITY, IA 52242

BURG, MATTHEW M, PHD
PROFESSOR
DEPARTMENT OF INTERNAL MEDICINE
YALE UNIVERSITY SCHOOL OF MEDICINE
NEW HAVEN, CT 06520

CHAYTOR, NAOMI S, PHD *
ASSOCIATE PROFESSOR
DEPARTMENT OF MEDICAL EDUCATION
AND CLINICAL SCIENCES
ELSON S. FLOYD COLLEGE OF MEDICINE
WASHINGTON STATE UNIVERISITY
SPOKANE, WA 99202

COLLOCA, LUANA, PHD *
ASSOCIATE PROFESSOR
DEPARTMENT OF PAIN TRANSLATIONAL SYMPTOM
SCIENCE
SCHOOL OF NURSING
UNIVERSITY OF MARYLAND
BALTIMORE, MD 21201

D'ALONZO, KAREN T, PHD *
ASSOCIATE PROFESSOR
DIVISION OF NURSING SCIENCE
SCHOOL OF NURSING
RUTGERS BIOMEDICAL AND HEALTH SCIENCES
RUTGERS, THE STATE UNIVERSITY OF NEW JERSEY
NEWARK, NJ 07102

DUNNE, EUGENE M, PHD *
ASSISTANT PROFESSOR
DEPARTMENT OF ORAL HEALTH SCIENCES
TEMPLE UNIVERSITY
PHILADELPHIA, PA 19140

FORTIER, MICHELLE, PHD
ASSOCIATE PROFESSOR
SUE AND BILL GROSS SCHOOL OF NURSING
UNIVERSITY OF CALIFORNIA, IRVINE
ORANGE, CA 92868

GALLO, LINDA C, PHD *
PROFESSOR
DEPARTMENT OF PSYCHOLOGY
SAN DIEGO STATE UNIVERSITY
SAN DIEGO, CA 92123

GOLDMAN, MYLA D, MD *
PROFESSOR AND VICE CHAIR
DEPARTMENT OF NEUROLOGY
SCHOOL OF MEDICINE
VIRGINIA COMMONWEALTH UNIVERSITY
RICHMOND, VA 23298

HARPER, FELICITY, PHD
ASSOCIATE PROFESSOR
DEPARTMENT OF ONCOLOGY
KARMANOS CANCER INSTITUTE
WAYNE STATE UNIVERSITY
DETROIT, MI 48201

HASSETT, AFTON L, PSYD
ASSOCIATE PROFESSOR
DEPARTMENT OF ANESTHESIOLOGY
CHRONIC PAIN AND FATIGUE RESEARCH CENTER
UNIVERSITY OF MICHIGAN MEDICAL SCHOOL
ANN ARBOR, MI 48106

KRONISH, IAN M, MD
ASSOCIATE PROFESSOR
DEPARTMENT OF MEDICINE
COLUMBIA UNIVERSITY MEDICAL CENTER
NEW YORK, NY 10032

MATHEW, JOSEPH P, MD
PROFESSOR
DEPARTMENT OF ANESTHESIOLOGY
DUKE UNIVERSITY MEDICAL CENTER
DURHAM, NC 27710

MEYER, JACOB D, PHD *
ASSISTANT PROFESSOR
DEPARTMENT OF KINESIOLOGY
COLLEGE OF HUMAN SCIENCES
IOWA STATE UNIVERSITY
AMES, IA 50011

NAPADOW, VITALY, PHD *
ASSOCIATE PROFESSOR
MARTINOS CENTER FOR BIOMEDICAL IMAGING
MASSACHUSETTS GENERAL HOSPITAL
HARVARD MEDICAL SCHOOL
CHARLESTOWN, MA 02129

PYATAK, ELIZABETH F, PHD *
ASSOCIATE PROFESSOR
DEPARTMENT OF OCCUPATIONAL SCIENCE
AND OCCUPATIONAL THERAPY
HEALTH SCIENCE CAMPUS
UNIVERSITY OF SOUTHERN CALIFORNIA
LOS ANGELES, CA 90089

RHEE, KYUNG E, MD
PROFESSOR
DEPARTMENT OF PEDIATRICS
UNIVERSITY OF CALIFORNIA, SAN DIEGO
LA JOLLA, CA 92093

RINI, CHRISTINE, PHD
PROFESSOR
DEPARTMENT OF MEDICAL SOCIAL SCIENCES
FEINBERG SCHOOL OF MEDICINE
NORTHWESTERN UNIVERSITY
CHICAGO, IL 60611

ROPER, JENEVIEVE L, PHD *
ASSISTANT PROFESSOR
DEPARTMENT OF HEALTH AND HUMAN SCIENCES
COLLEGE OF SCIENCE AND ENGINEERING
LOYOLA MARYMOUNT UNIVERSITY
LOS ANGELES, CA 90045

SALWEN-DEREMER, JESSICA K, PHD *
ASSISTANT PROFESSOR
PSYCHIATRY AND MEDICINE
GEISEL SCHOOL OF MEDICINE
DARTMOUTH COLLEGE
HANOVER, NH 03755

SCHEMBRE, SUSAN, PHD *
ASSOCIATE PROFESSOR
DEPARTMENT OF FAMILY AND COMMUNITY MEDICINE
COLLEGE OF MEDICINE TUCSON
THE UNIVERSITY OF ARIZONA HEALTH SCIENCES
TUCSON, AZ 85714

SCHENKER, YAEL, MD *
PROFESSOR OF MEDICINE
SECTION OF PALLIATIVE CARE AND MEDICAL ETHICS
DIVISION OF GENERAL INTERNAL MEDICINE
UNIVERSITY OF PITTSBURGH
PITTSBURGH, PA 15261

SCIAMANNA, CHRISTOPHER N, MD, MPH *
PROFESSOR OF MEDICINE AND PUBLIC HEALTH SERVICES
DIVISION OF GENERAL INTERNAL MEDICINE
MILTON S HERSHEY COLLEGE OF MEDICINE
PENNSYLVANIA STATE UNIVERSITY
HERSHEY, PA 17033

SEN, SOUVIK, MD, MPH *
PROFESSOR AND CENTER FOR ECONOMIC EXCELLENCE
STROKE CHAIR
DEPARTMENT OF NEUROLOGY
UNIVERSITY OF SOUTH CAROLINA
COLUMBIA, SC 29203

SMITH, PATRICK J, PHD
ASSOCIATE PROFESSOR
DEPARTMENT OF PSYCHIATRY AND BEHAVIORAL
SCIENCES
MEDICINE AND POPULATION HEALTH SCIENCES
DUKE UNIVERSITY
DURHAM, NC 27710

SMITH, SAKIMA A, MD *
ASSOCIATE PROFESSOR
DIVISION OF CARDIOVASCULAR MEDICINE
DEPARTMENT OF INTERNAL MEDICINE
WEXNER MEDICAL CENTER
THE OHIO STATE UNIVERSITY
COLUMBUS, OH 43210

TEMPLE, JENNIFER L, PHD
PROFESSOR
DEPARTMENTS OF EXERCISE AND NUTRITION SCIENCES
AND COMMUNITY HEALTH AND HEALTH BEHAVIOR
SCHOOL OF PUBLIC HEALTH AND HEALTH PROFESSIONS
UNIVERSITY AT BUFFALO
BUFFALO, NY 14214

UEBELACKER, LISA A, PHD
PROFESSOR
DEPARTMENT OF PSYCHIATRY AND HUMAN BEHAVIOR
BUTLER HOSPITAL
BROWN UNIVERSITY
PROVIDENCE, RI 02906

VAN CLEAVE, JANET H, PHD *
ASSISTANT PROFESSOR
RORY MEYERS COLLEGE OF NURSING
NEW YORK UNIVERSITY
NEW YORK, NY 10010

VOLLMER, TIMOTHY LEE, MD *
PROFESSOR
DEPARTMENT OF NEUROLOGY
ROCKY MOUNTAIN MS CLINIC ANSCHUTZ MEDICAL CENTER
SCHOOL OF MEDICINE
UNIVERSITY OF COLORADO DENVER
AURORA, CO 80045

WEN, KUANG-YI, PHD *
ASSOCIATE PROFESSOR
SIDNEY KIMMEL CANCER CENTER
THOMAS JEFFERSON UNIVERSITY
PHILADELPHIA, PA 19107

MAIL REVIEWER(S)

PALESH, OXANA G, PHD
ASSOCIATE PROFESSOR
DEPARTMENT OF PSYCHIATRY AND BEHAVIORAL
SCIENCES
STANFORD UNIVERSITY SCHOOL OF MEDICINE
STANFORD, CA 94305

SCIENTIFIC REVIEW OFFICER

VOSVICK, MARK A, PHD
SCIENTIFIC REVIEW OFFICER
CENTER FOR SCIENTIFIC REVIEW
NATIONAL INSTITUTES OF HEALTH
BETHESDA, MD 20892

EXTRAMURAL SUPPORT ASSISTANT

WATTS, MELISSA D
EXTRAMURAL SUPPORT ASSISTANT
CENTER FOR SCIENTIFIC REVIEW
NATIONAL INSTITUTE FOR HEALTH
BETHESDA, MD 20892

* Temporary Member. For grant applications, temporary members may participate in the entire meeting or may review only selected applications as needed.

Consultants are required to absent themselves from the room during the review of any application if their presence would constitute or appear to constitute a conflict of interest.
